# Dose-Dependent Cellular Phenotypic Change Induced by ^177^Lu-Oxodotreotide Treatment in IMR-32 Cells

**DOI:** 10.3390/biomedicines13071543

**Published:** 2025-06-25

**Authors:** Shuai Xue, Xiaobei Zheng, Bingbing Pu, Xiao Li, Jun Li, Meng Huang, Jian Yang, Jingjing Lou

**Affiliations:** 1 Department of Nuclear Medicine, Shanghai Pudong Hospital, Fudan University Pudong Medical Center, Shanghai 201399, China; xueshuai@fudan.edu.cn (S.X.); lixiao@sinap.ac.cn (X.L.); lijunlx@foxmail.com (J.L.);; 2 Shanghai Institute of Applied Physics, Chinese Academy of Sciences, Shanghai 201800, China; zhengxiaobei@sinap.ac.cn; 3 Department of Rehabilitation Medicine, Shanghai Pudong Hospital, Fudan University Pudong Medical Center, Shanghai 201399, China; pubingbing91@163.com; 4 Department of Nuclear Medicine, Shanghai Changhai Hospital, Shanghai 200433, China

**Keywords:** ^177^Lu-Oxodotreotide, β-emitting therapeutics, cell damage, dose dependent

## Abstract

**Objectives**: Beta-emitting radionuclide therapy, exemplified by ^177^Lu-Oxodotreotide (Lutathera^®^), enables targeted treatment of neuroendocrine tumors by delivering β-radiation to tumor cells. However, the dose-dependent molecular mechanisms underlying cellular damage remain insufficiently characterized. This study aimed to investigate the phenotypic changes in IMR-32 human neuroblastoma cells following Lutathera exposure, with a focus on the dose-dependent relationship between radiation and cellular damage. **Methods**: IMR-32 cells were allocated to control, low- (0.05 MBq/mL), medium- (0.5 MBq/mL), and high-dose (5 MBq/mL) groups and treated with ^177^Lu-Oxodotreotide for 24 h. Flow cytometry was employed to assess cell viability, apoptosis, mitochondrial membrane potential, γ-H2AX expression (a marker of DNA damage), and proliferation. **Results**: Lutathera induced dose-dependent cytotoxic effects. Cell viability declined linearly with increasing dose (control: 100% vs. high-dose: 13.48%; r = −0.955, *p* < 0.001). Apoptosis was significantly elevated (control: 35.34% vs. high-dose: 88.12%; r = 0.999), accompanied by increased γ-H2AX levels (control: 5.26 × 10^4^ vs. high-dose: 13.13 × 10^4^; r = 0.930), indicating DNA double-strand breaks. Mitochondrial membrane potential decreased (control: 6.06 × 10^4^ vs. high-dose: 46.27 × 10^4^; r = 0.999), and proliferation was suppressed (control: 91.10 × 10^4^ vs. high-dose: 103.84 × 10^4^; r = 0.954), both showing strong dose correlations (*p* < 0.001). **Conclusions**: ^177^Lu-Oxodotreotide exerts dose-dependent cytotoxicity in IMR-32 cells via DNA damage, mitochondrial dysfunction, and apoptosis induction. These findings underscore the necessity of optimizing dosing regimens to balance therapeutic efficacy and safety in clinical settings, providing a foundation for personalized β-emitter therapies.

## 1. Introduction

Radiotherapeutic drugs are a class of agents that selectively deliver cytotoxic radionuclides to lesion sites, where the radiation emitted from radionuclide decay exerts cytotoxic effects on target cells to achieve therapeutic outcomes [1]. The principles guiding dosage selection differ fundamentally depending on the type of therapeutic nuclide. Alpha (α)-emitting radionuclides are typically considered dose-independent due to their ability to directly induce DNA damage in cells [2,3,4,5,6]. In contrast, beta (β)-emitting radionuclides exert indirect cytotoxicity by promoting the generation of free radicals, and their therapeutic efficacy is closely correlated with the administered dose [7,8,9]. Moreover, β-emitter-induced cytotoxicity involves diverse mechanisms, including modulation of signaling pathways and cell cycle arrest, which remain to be fully elucidated. Understanding the dose-dependent mechanisms of cellular injury is essential for the rational optimization of β-emitter dosing regimens.

In recent years, β-emitting therapeutic agents, particularly those based on ^177^Lu, have advanced rapidly and enabled targeted precision therapy for a wide range of diseases [10,11,12,13]. Among these, ^177^Lu-Oxodotreotide (^177^Lu-DOTATATE, Lutathera^®^) is a radioligand therapy (RLT) that facilitates the precision treatment of neuroendocrine neoplasms (NENs) by selectively binding to somatostatin receptor 2 (*SSTR2*) [14,15,16]. This mechanism relies on the high-affinity binding of the ^177^Lu-DOTATATE radiocomplex to *SSTR2*, wherein the somatostatin analogue moiety enables targeted delivery of the β-emitting ^177^Lu radionuclide to tumor sites. The emitted β-particle radiation induces both single- and double-stranded DNA breaks, ultimately leading to apoptosis [17]. Findings from the phase III NETTER-1 clinical trial demonstrated that peptide receptor radionuclide therapy (PRRT) with ^177^Lu-Oxodotreotide was significantly more effective than high-dose, long-acting octreotide in patients with well-differentiated grade 1 and 2 midgut NENs. PRRT resulted in substantial improvements in progression-free survival (PFS) and overall survival (OS) [18]. Based on these results, the U.S. Food and Drug Administration and the European Medicines Agency have approved ^177^Lu-Oxodotreotide for the treatment of *SSTR2*-positive gastroenteropancreatic neuroendocrine tumors in patients aged 12 years and older. Clinical studies have further confirmed its ability to significantly prolong PFS and enhance quality of life [19,20]. However, most available studies have focused on evaluating the efficacy and safety of fixed clinical dosing regimens (e.g., 7.4 GBq every 8 weeks for four cycles), while the molecular mechanisms underlying dose-dependent effects remain insufficiently understood. Notably, in vitro studies using IMR-32 human neuroblastoma cells lack systematic data. Therefore, this study aimed to elucidate the dose-dependent cytotoxic effects of Lutathera in IMR-32 cells through in vitro experiments, thereby providing a theoretical foundation for the optimization of individualized treatment regimens.

## 2. Materials and Methods

### 2.1. Human Neuroblastoma Cell (IMR-32) Culture and Processing

IMR-32 human neuroblastoma cells were obtained from Shanghai Chuanqiu Biotechnology Co., Ltd. (Shanghai, China). Cells were cultured in 100 mm cell culture dishes using Dulbecco’s modified eagle medium (DMEM; Adamas life, Titan, Shanghai, China) supplemented with 10% fetal bovine serum (FBS; Gibco, Grand Island, NE, USA), 100 U/mL penicillin, and 100 U/mL streptomycin (Shanghai Beyotime Biotechnology Co., Ltd., Shanghai, China). Cultures were maintained at 37 °C in a humidified atmosphere containing 5% CO_2_ and 95% air. The medium was replaced every two days, and cells were passaged using 0.25% trypsin–EDTA (Adamas Life, Titan, Shanghai, China).

Cells were divided into four groups: control, low-dose (0.05 MBq/mL), medium-dose (0.5 MBq/mL), and high-dose (5 MBq/mL). For treatment, 1 × 10^6^ cells were seeded in 6-well plates with 3 mL of complete medium per well. After overnight incubation (37 °C, 5% CO_2_), the medium was replaced, and cells were exposed to the designated dose of ^177^Lu-Oxodotreotide for 24 h.

### 2.2. Synthesis of ^177^Lu-Oxodotreotide

^177^Lu-Oxodotreotide was synthesized by sequentially adding ^177^LuCl_3_ (Isotopia Molecular Imaging Ltd., Petach Tikva, Israel) solution (3 μL, 100 MBq), DOTATATE (12 μL, 2.65 nmol; prepared in 0.5 M acetic acid–sodium acetate buffer, pH 5.2), and gentisic acid (15 μL, 974 nmol) into a reaction flask. The mixture was gently mixed and incubated at 95 °C for 15 min. After incubation, the solution was cooled to room temperature and diluted with 120 μL of sodium ascorbate solution (0.3 M). The radiochemical purity (RCP) of the product was evaluated using radio high-performance liquid chromatography (radio-HPLC) (IdealChrom 910, ELAY Technologies, Inc., Shanghai, China) [A: CH_3_CN-0.1% trifluoroacetic acid (TFA), B: H_2_O-0.1% TFA, 25–50–100% A (0–20–20.1 min)]. The wavelength, column temperature, flow rate, and sample volume were set to 220 nm, 30 °C, 1 mL/min, and 20 μL, respectively. ^177^Lu-Oxodotreotide with > 95% RCP was used for the cell experiments.

### 2.3. Stability Test

Samples were mixed with 1 × phosphate-buffered saline (PBS, Gibco, Grand Island, NE, USA) or 1% FBS and incubated at room temperature (RT) to evaluate the stability of ^177^Lu-Oxodotreotide. Samples were taken at 0, 24, and 36 h (n = 3) and analyzed using radio-HPLC [A: CH_3_CN-0.1% TFA, B: H_2_O-0.1% TFA, 25–50–100% A (0–20–20.1 min), 1 mL/min].

### 2.4. Cell Viability Assay

We assessed cell viability by using a 7-amino-actinomycin D (7-AAD) staining kit according to the manufacturer’s instructions (Shanghai Beyotime Biotechnology Co., Ltd., Shanghai, China). Upon excitation with a 488 nm laser, 7-AAD emits fluorescence detected in the far-red range (≥650 nm, long-pass filter). The dye selectively penetrates late apoptotic and necrotic cells with compromised membranes, binding to nuclear DNA. Cells from each treatment group were collected and prepared as single-cell suspensions. A total of 1 × 10^6^ cells per group were centrifuged at 600× *g* for 5 min, after which the supernatant was discarded. The cell pellets were resuspended in 1 mL of 7-AAD staining solution and incubated at 37 °C in the dark for 10 min. After incubation, cells were washed twice with 1 mL of cold 1× PBS and resuspended in 1 mL 1× PBS for flow cytometry analysis. Samples were analyzed using a NovoCyte flow cytometer (NovoCyte 2060R, Agilent Biosciences, San Diego, CA, USA) and NovoExpress software (version 1.6.2, Agilent Biosciences, San Diego, CA, USA). At least three biological replicates were assessed per group. Mean fluorescence intensity (MFI) was used as the quantitative indicator of cell viability.

### 2.5. Cell Division Rate Assay

The rate of cell division was assessed using the carboxyfluorescein diacetate succinimidyl ester (CFDA SE) labeling method (CFDA SE Cell Proliferation and Tracer Assay Kit, Shanghai Beyotime Biotechnology Co., Ltd., Shanghai, China), following the manufacturer’s instructions. Briefly, 1 × 10^6^ cells were incubated with 2 mL of CFDA SE in a 15 mL centrifuge tube for 15 min at 37 °C. Following incubation, 10 mL of medium containing 10% FBS was added, and the suspension was centrifuged at 1000× *g* for 5 min. This washing step was repeated twice to remove excess dye. Approximately 1 × 10^5^ labeled IMR-32 cells were seeded into 6-well plates and treated with ^177^Lu-Oxodotreotide for 24 h. Fluorescence imaging was performed using a Nexcope fluorescence microscope (Ningbo Yongxin Optics Co., Ltd., Ningbo, China). Samples were subsequently analyzed by flow cytometry (NovoCyte 2060R, Agilent Biosciences, San Diego, CA, USA), and data processing was conducted using NovoExpress software (version 1.6.2, Agilent Biosciences, San Diego, CA, USA). MFI was used to quantify changes in the cell division rate.

### 2.6. Cell Apoptosis Assay

Cell apoptosis was assessed by flow cytometry using the Annexin V–fluorescein isothiocyanate (FITC)/propidium iodide (PI) Apoptosis Detection Kit (Shanghai Beyotime Biotechnology Co., Ltd., Shanghai, China), following the manufacturer’s protocol. IMR-32 cells treated with ^177^Lu-Oxodotreotide were digested, collected, and centrifuged at 1000× *g* for 5 min. The cell pellets were washed once with cold PBS and centrifuged again under the same conditions. Each sample was resuspended in 195 μL of 1× Binding Buffer, followed by the addition of 5 μL Annexin V-FITC and 10 μL PI. After 15 min of incubation at RT in the dark, an additional 300 μL of 1× Binding Buffer was added. Samples were analyzed by flow cytometry within 15 min of staining.

### 2.7. Mitochondrial Membrane Potential Assay

To evaluate early apoptosis, mitochondrial membrane potential was assessed using the JC-10 Mitochondrial Membrane Potential Detection Kit (Biosharp Life Sciences, Shanghai, China) according to the manufacturer’s instructions. Cells from each group were collected and centrifuged at 600× *g* for 5 min. The cell pellets were resuspended in 0.5 mL of complete culture medium, followed by the addition of 0.5 mL JC-10 working solution. The suspension was gently mixed by inversion and incubated at 37 °C for 20 min. During incubation, JC-10 staining buffer (5×) was diluted with distilled water at a ratio of 1:4 (e.g., 4 mL water per 1 mL buffer) and kept on ice. After incubation, cells were centrifuged at 600× *g* for 3 min at 4 °C, and the supernatant was discarded. Cells were then washed twice with 1× JC-10 staining buffer. The resulting pellet was resuspended in 1 mL of 1× JC-10 staining buffer and centrifuged again under the same conditions, and the supernatant was discarded. Finally, cells were resuspended in 500 μL of 1× JC-10 staining buffer and analyzed immediately by flow cytometry. JC-10 monomers, indicating mitochondrial depolarization, were detected via the FITC channel (green fluorescence), and the MFI of green fluorescence was used to quantify changes in mitochondrial membrane potential.

### 2.8. DNA Damage Assay

DNA double-strand break (DSB) damage was evaluated by measuring the phosphorylation of H2AX at Ser139 (γ-H2AX) using flow cytometry. Cells from each group were harvested, washed with PBS, and fixed with 1% paraformaldehyde. After fixation, cells were washed again and permeabilized with 70% ethanol at −20 °C. Following permeabilization, ethanol was removed, and the cells were washed with PBS. A PE-conjugated anti-γ-H2AX antibody (#12-9865-42, Thermo Fisher Scientific, Waltham, MA, USA) was then added, and samples were incubated for 30 min at room temperature in the dark. After staining, samples were immediately analyzed by flow cytometry, and at least 10,000 events were collected per sample.

### 2.9. Statistical Analysis

All of the data are presented as mean ± standard error of the mean. Statistical significance was assessed using one-way analysis of variance (ANOVA), followed by Dunnett’s multiple comparison test. A *p*-value of less than 0.05 was considered statistically significant. Data analysis was performed using SPSS software (version 21.0, SPSS Inc., Chicago, IL, USA) and Origin 2024b (OriginLab, Northampton, MA, USA).

## 3. Results

### 3.1. Synthesis and In Vitro Stability of ^177^Lu-Oxodotreotide

**Figure 1:**
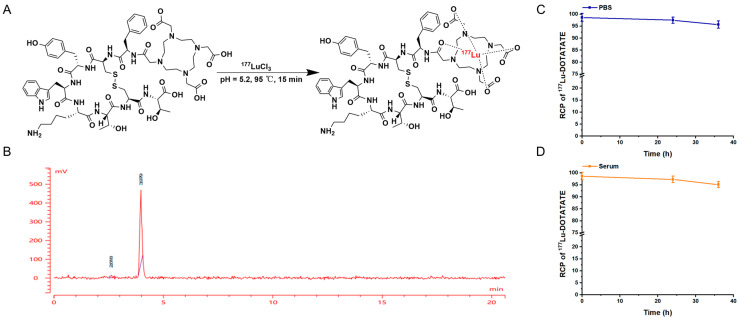
(**A**) Synthesis of ^177^Lu-Oxodotreotide. (**B**) Radio-HPLC spectrum showing the radioactive peak of ^177^Lu-Oxodotreotide. Radiochemical purity (RCP) of ^177^Lu-Oxodotreotide measured over 36 h in phosphate-buffered saline (PBS) (**C**) and fetal bovine serum (FBS) (**D**).

Figure 1A illustrates the coordination interaction between DOTA and ^177^Lu during the synthesis of ^177^Lu-Oxodotreotide. The RCP of ^177^Lu-Oxodotreotide was 98.67% ± 1.59% (n = 3), as determined by radio-HPLC analysis (Figure 1B). The UV absorption peak was observed at 5.75 min, which corresponded closely to the radioactive peak (Figure 1B) detected at a similar retention time. The stability of ^177^Lu-Oxodotreotide in PBS and serum was evaluated, with results indicating an RCP greater than 95% over a 36 h observation period (Figure 1C,D). These findings demonstrate that ^177^Lu-Oxodotreotide exhibits high in vitro stability.

### 3.2. Changes in Cell Viability After Treatment with Different Doses of ^177^Lu-Oxodotreotide

**Figure 2:**
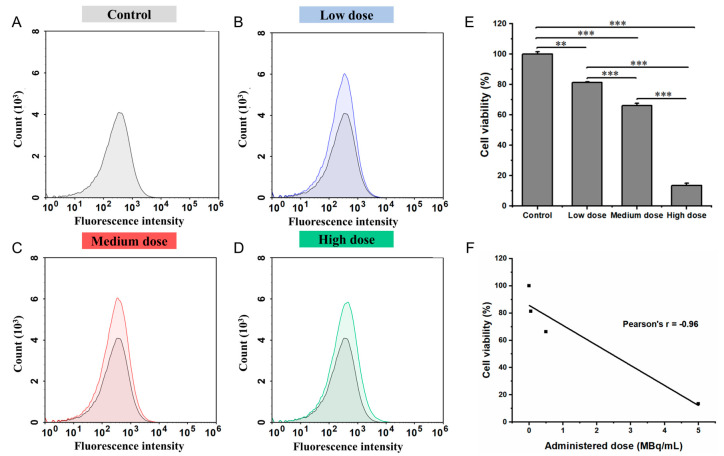
Histograms showing superimposed signals of cell viability in control group (**A**), control and low-dose group (**B**), control and medium-dose group (**C**), and control and high-dose group (**D**). (**E**) Comparison of cell viability among experimental groups, measured by MFI. Data are presented as mean ± SD; ** *p* < 0.01, *** *p* < 0.001. (**F**) Linear correlation between administered dose of ^177^Lu-Oxodotreotide and IMR-32 cell viability.

The effects of varying doses of ^177^Lu-Oxodotreotide on IMR-32 cell viability are shown in Figure 2. After 24 h incubation with gradually increasing concentrations of ^177^Lu-oxodotreotide, the survival rate of IMR-32 cells significantly decreased in a dose-dependent manner compared to the control group (Figure 2A). Specifically, viability decreased from 100.00 ± 1.59% in the control group to 81.41 ± 0.33% in the low-dose group (*p* < 0.01; Figure 2B), 66.23 ± 1.39% in the medium-dose group (*p* < 0.001; Figure 2C), and 13.48 ± 1.47% in the high-dose group (*p* < 0.001; Figure 2D). As shown in Figure 2E, the inhibitory effect on IMR-32 cell viability increased with higher ^177^Lu-Oxodotreotide doses. A strong inverse linear correlation was observed between the administered dose and the MFI of cell viability (r = −0.96; Figure 2F), indicating dose-dependent cytotoxicity.

### 3.3. Changes in Cell Division After Treatment with Different Doses of ^177^Lu-Oxodotreotide

**Figure 3:**
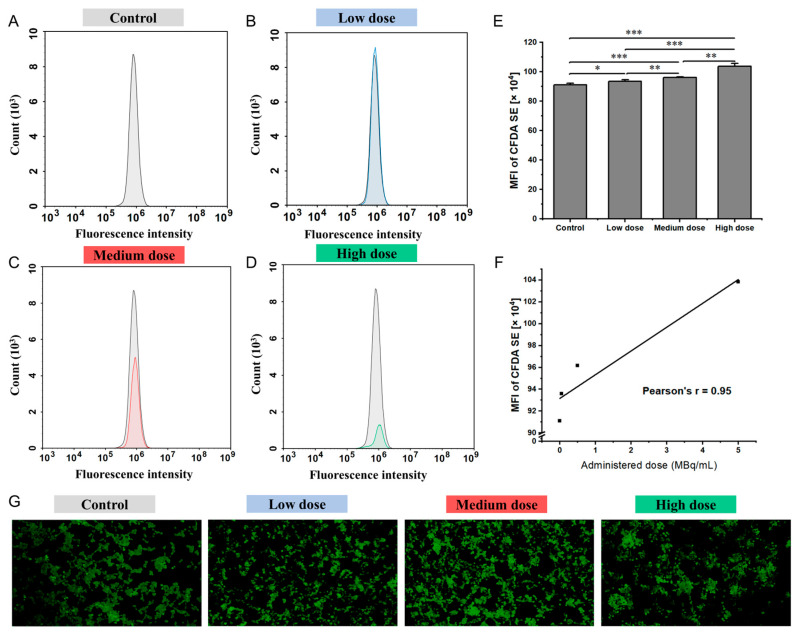
Histograms showing superimposed signals of cell proliferation in control group (**A**), control and low-dose group (**B**), control and medium-dose group (**C**), and control and high-dose group (**D**). (**E**) Comparison of cell proliferation among experimental groups, measured by MFI. Data are presented as mean ± SD; * *p* < 0.05, ** *p* < 0.01, *** *p* < 0.001. (**F**) Linear correlation between administered dose of ^177^Lu-Oxodotreotide and IMR-32 cell proliferation. (**G**) Representative fluorescence images showing proliferation differences among groups.

The effects of varying doses of ^177^Lu-oxodotreotide on IMR-32 cell proliferation are shown in Figure 3. Compared with the control group (Figure 3A), IMR-32 cells exhibited a gradual and statistically significant increase in MFI following treatment with escalating doses of ^177^Lu-Oxodotreotide: low-dose (91.10 ± 1.02 × 10^4^ vs. 93.59 ± 1.12 × 10^4^, control vs. low-dose group, *p* < 0.05; Figure 3B), medium-dose (91.10 ± 1.02 × 10^4^ vs. 96.18 ± 0.31 × 10^4^, *p* < 0.001; Figure 3C), and high-dose (91.10 ± 1.02 × 10^4^ vs. 103.84 ± 1.85 × 10^4^, *p* < 0.001; Figure 3D). As shown in Figure 3E, this dose-dependent increase in MFI reflects a decrease in the cell division rate, as cells undergoing fewer divisions accumulate more fluorescence. As can be seen in Figure 3F, a strong linear correlation was observed between the ^177^Lu-Oxodotreotide dose and MFI (r = 0.95), indicating a consistent inhibitory effect on cell proliferation. Figure 3G illustrates that fluorescence intensity increased progressively with higher doses, further confirming reduced cell division and proliferation in IMR-32 cells at elevated treatment levels.

### 3.4. Changes in Cell Apoptosis After Treatment with Different Doses of ^177^Lu-Oxodotreotide

**Figure 4:**
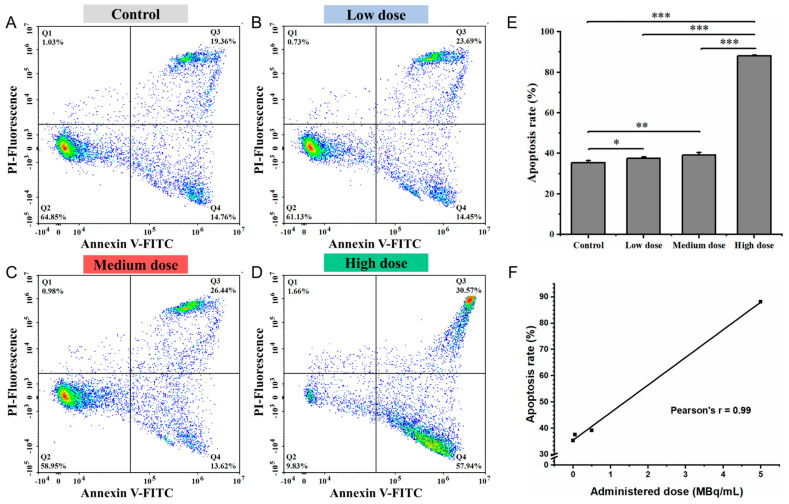
Apoptosis induced by ^177^Lu-Oxodotreotide was evaluated using Annexin V-FITC/PI staining and flow cytometry. Representative FITC/PI bivariate density plots are shown for control group (**A**), low-dose group (**B**), medium-dose group (**C**), and high-dose group (**D**). Cells were classified as healthy cells (Annexin V^−^, PI^−^), early apoptotic cells (Annexin V^+^, PI^−^), late apoptotic cells (Annexin V^+^, PI^+^), or damaged cells (Annexin V^−^, PI^+^). (**E**) Comparison of apoptosis rates across experimental groups, calculated as the sum of early and late apoptotic percentages. Data are presented as mean ± SD; * *p* < 0.05, ** *p* < 0.01, *** *p* < 0.001. (**F**) Linear correlation between apoptosis rate and administered dose of ^177^Lu-Oxodotreotide.

The apoptotic response of IMR-32 cells following treatment with various doses of ^177^Lu-Oxodotreotide is presented in Figure 4. Compared with the control group (Figure 4A), apoptosis rates increased significantly and progressively in response to increasing treatment doses. Specifically, apoptosis rose from 35.34 ± 1.08% in the control group to 37.51 ± 0.84% in the low-dose group (*p* < 0.05; Figure 4B), 39.20 ± 1.23% in the medium-dose group (*p* < 0.01; Figure 4C), and 88.12 ± 0.36% in the high-dose group (*p* < 0.001; Figure 4D). As shown in Figure 4E, apoptosis levels increased at higher ^177^Lu-Oxodotreotide concentrations. A strong positive linear correlation was observed between the administered dose and the apoptosis rate (r = 0.99; Figure 4F), confirming a robust dose-dependent pro-apoptotic effect of ^177^Lu-Oxodotreotide on IMR-32 cells.

### 3.5. Changes in Mitochondrial Membrane Potential After Treatment with Different Doses of ^177^Lu-Oxodotreotide

**Figure 5:**
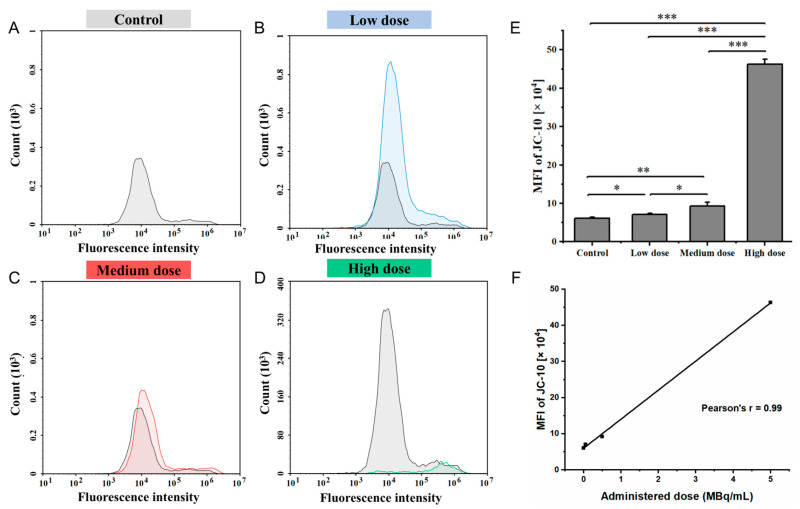
Histograms showing superimposed signals of mitochondrial membrane potential in control group (**A**), control and low-dose group (**B**), control and medium-dose group (**C**), and control and high-dose group (**D**). (**E**) Comparison of mitochondrial membrane potential across experimental groups, evaluated by MFI. Data are presented as mean ± SD; * *p* < 0.05, ** *p* < 0.01, *** *p* < 0.001. (**F**) Linear correlation between administered dose of ^177^Lu-Oxodotreotide and mitochondrial membrane potential in IMR-32 cells.

Changes in mitochondrial membrane potential in IMR-32 cells following exposure to different doses of ^177^Lu-Oxodotreotide are shown in Figure 5. Compared with the control group (Figure 5A), a dose-dependent increase in FITC fluorescence intensity was observed after ^177^Lu-Oxodotreotide treatment, indicating a loss of mitochondrial membrane potential. Specifically, fluorescence intensity increased in the low-dose group (6.06 ± 0.30 × 10^4^ vs. 7.07 ± 0.35 × 10^4^, control vs. low-dose, *p* < 0.05; Figure 5B), the medium-dose group (6.06 ± 0.30 × 10^4^ vs. 9.25 ± 0.99 × 10^4^, *p* < 0.01; Figure 5C), and the high-dose group (6.06 ± 0.30 × 10^4^ vs. 46.27 ± 1.32 × 10^4^, *p* < 0.001; Figure 5D). As shown in Figure 5E, the increase in FITC fluorescence intensity with higher doses reflects the failure of JC-10 dye to aggregate within the mitochondrial matrix, indicating a significant reduction in mitochondrial membrane potential. A strong positive linear correlation was observed between the administered dose and the measured change in mitochondrial membrane potential (r = 0.99; Figure 5F), confirming a dose-dependent mitochondrial dysfunction induced by ^177^Lu-Oxodotreotide.

### 3.6. Changes in DNA Damage After Treatment with Different Doses of ^177^Lu-Oxodotreotide

**Figure 6:**
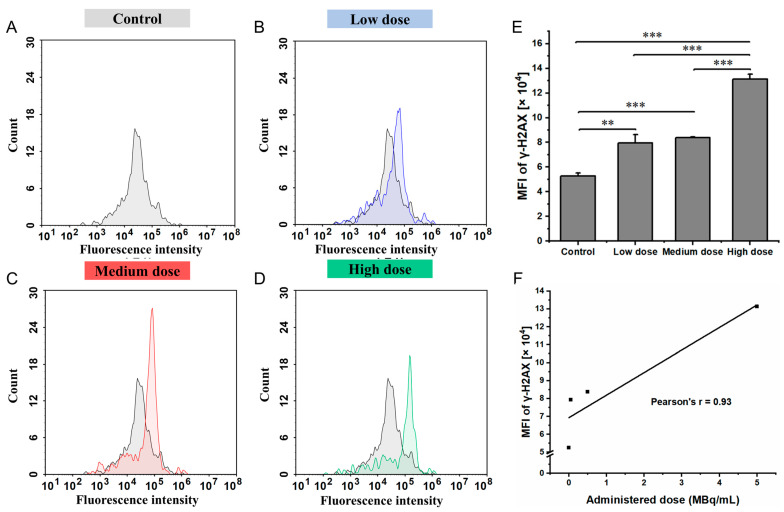
Histograms showing superimposed signals of DNA damage in control group (**A**), control and low-dose group (**B**), control and medium-dose group (**C**), and control and high-dose group (**D**) cells. (**E**) Comparison of DNA damage levels quantified by γ-H2AX MFI across experimental groups. Data are presented as mean ± SD; ** *p* < 0.01, *** *p* < 0.001. (**F**) Linear correlation between administered dose of ^177^Lu-Oxodotreotide and DNA damage (γ-H2AX accumulation) in IMR-32 cells.

The accumulation of γ-H2AX in IMR-32 cells, indicative of DNA double-strand breaks, following treatment with various doses of ^177^Lu-oxodotreotide is shown in Figure 6. Compared with the control group (Figure 6A), intracellular γ-H2AX levels increased progressively in a dose-dependent manner, with statistically significant differences observed across groups. Specifically, γ-H2AX accumulation increased in the low-dose group (5.26 ± 0.25 × 10^4^ vs. 7.94 ± 0.70 × 10^4^, control vs. low-dose, *p* < 0.05; Figure 6B), medium-dose group (5.26 ± 0.25 × 10^4^ vs. 8.38 ± 0.06 × 10^4^, *p* < 0.001; Figure 6C), and high-dose group (5.26 ± 0.25 × 10^4^ vs. 13.13 ± 0.39 × 10^4^, *p* < 0.001; Figure 6D). As shown in Figure 6E, γ-H2AX accumulation increased significantly with higher doses of ^177^Lu-Oxodotreotide, indicating enhanced DNA damage. A strong positive linear correlation was observed between the administered dose and γ-H2AX levels (r = 0.93; Figure 6F), confirming a dose-dependent induction of DNA double-strand breaks in IMR-32 cells.

## 4. Discussion

Although radiotherapeutic drugs in the preclinical stage share similarities with conventional drug development pathways, their complex mechanisms of action necessitate tailored research strategies to ensure accuracy and effectiveness. Early-phase development typically begins with in vitro studies, which assess physicochemical characteristics and cellular-level parameters such as affinity, specificity, metabolism, and serum stability. Based on these findings, animal studies are conducted to further evaluate drug efficacy and safety. Subsequently, data from both in vitro and in vivo studies are integrated to identify and validate potential candidate molecules. These candidates are then advanced to clinical trials for comprehensive evaluation of therapeutic efficacy and safety.

The dose-dependent phenotypic changes observed in IMR-32 cells treated with ^177^Lu-Oxodotreotide underscore the distinct cellular mechanisms activated at varying radiation intensities. At low-dose irradiation (0.05 MBq/mL), the primary response was the inhibition of cell proliferation (MFI: 91.10 × 10^4^ vs. 93.59 × 10^4^, *p* < 0.05), consistent with the characterization of low-dose β-radiation as predominantly inducing sub-lethal DNA single-strand breaks [21]. Cell proliferation involves the division of cells to generate progeny, and radiation from radionuclide therapy can hit cellular DNA, leading to various forms of damage, including base modifications, single-strand breaks, and double-strand breaks. Consequently, ^177^Lu-Oxodotreotide may suppress tumor growth by disrupting cell cycle progression and preventing the successful division of malignant cells [22]. Emerging evidence suggests that such damage may be associated with inactivation of the ATM-mediated DNA repair pathway, resulting in sustained activation of cell cycle checkpoints, such as G2/M phase arrest [21]. Interestingly, apoptosis was only modestly increased at this dose level (35.34% vs. 37.51%, *p* < 0.05), indicating that cells may initially favor DNA repair pathways over apoptosis. This adaptive response may involve upregulation of anti-apoptotic proteins such as Bcl-2, potentially mediated by pro-survival signaling via the Wnt/β-catenin pathway [23,24]. These findings suggest that under low radiation stress, IMR-32 cells may prioritize repair and survival rather than undergoing programmed cell death.

The high-dose group (5 MBq/mL) represented a transition point from repairable damage to irreversible apoptosis. An increase in γ-H2AX levels (5.26 ± 0.25 × 10^4^ vs. 13.13 ± 0.39 × 10^4^, *p* < 0.001) suggests functional impairment of the non-homologous end-joining (NHEJ) DNA repair pathway, while the observed decline in mitochondrial membrane potential (6.06 ± 0.30 × 10^4^ vs. 46.27 ± 1.32 × 10^4^, *p* < 0.001) indicates activation of the apoptotic execution phase [21,25]. This response is consistent with the “inverse dose-rate effect,” wherein sustained low-dose-rate irradiation saturates the DNA repair machinery, leading to the accumulation of otherwise repairable lesions into lethal double-strand breaks [26,27]. This dose threshold may also be closely associated with the activation of the p53-dependent apoptotic pathway, reflecting a cellular shift from repair to programmed cell death once damage exceeds the repair capacity [28].

At high-dose exposure (5 MBq/mL), extensive apoptosis (88.12%, *p* < 0.001) and pronounced mitochondrial depolarization (46.27 × 10^4^, *p* < 0.001) were observed, alongside γ-H2AX accumulation (13.13 × 10^4^). These findings indicate that high-dose β-radiation overwhelms cellular defense mechanisms, leading to catastrophic DNA damage and mitochondrial collapse, which collectively drive late-stage apoptotic execution [29]. The strong linear correlations between the administered dose and both apoptosis (r = 0.99) and mitochondrial dysfunction (r = 0.99) underscore that cytotoxicity at this level is primarily driven by cumulative radiation-induced oxidative stress and direct DNA fragmentation. While the dose–response trends visually suggest monotonically increasing effects, the restricted dose cohorts represent a methodological limitation for quantitative analysis. This limitation reflects the complex logic of the ^177^Lu-Oxodotreotide study and requires further research. Nevertheless, the directional consistency across apoptosis (Figure 4), mitochondrial membrane potential (Figure 5), and DNA damage (Figure 6) provides biological validation. While de Jong’s work highlights membrane retention of *SSTR2* antagonists [15,30,31], ^177^Lu-Oxodotreotide—as a well-established agonist—undergoes rapid receptor-mediated internalization [32]. The high apoptosis rate (88.12% ± 0.36%) that we observed with the high dose may result from perinuclear DNA damage caused by internalized ^177^Lu, which may be related to the ‘crossfire effect’ of β-emitters. In addition, our data reveal a threshold effect (significant γ-H2AX elevation at ≥0.5 MBq/mL) that contextualizes de Jong’s reports of suboptimal tumor uptake at low ligand concentrations.

These dose-specific patterns suggest the existence of a therapeutic window: lower doses may be sufficient to exert cytostatic effects in slow-growing tumors, whereas higher doses are required to eliminate more aggressive malignancies. However, the steep increase in apoptosis and mitochondrial dysfunction at high-dose exposure also highlights the potential for off-target toxicity, underscoring the importance of precision in clinical dosing strategies. Future studies should investigate combinatorial approaches that either enhance the efficacy of low-dose treatments or reduce the adverse effects associated with high-dose regimens, building upon the mechanistic insights revealed in this study.

## 5. Conclusions

This study demonstrates that Lutathera (^177^Lu-Oxodotreotide) induces dose-dependent cytotoxic effects in IMR-32 neuroblastoma cells through multiple mechanisms, including DNA double-strand breaks, mitochondrial depolarization, and apoptosis activation. The observed linear correlations between radiation dose and phenotypic responses highlight the importance of precise dosage in maximizing therapeutic efficacy while minimizing off-target toxicity. While this in vitro model offers valuable mechanistic insights, further in vivo studies and clinical validation are essential to translate these findings into optimized treatment protocols. Overall, the results contribute to a deeper understanding of β-emitter therapeutics and support the development of individualized dosing strategies for neuroendocrine tumors.

## Data Availability

All data generated or analyzed during this study are included in this published article.

## Abbreviations

The following abbreviations are used in this manuscript:

RLT	Radioligand therapy
NENs	Neuroendocrine neoplasms
*SSTR2*	Somatostatin receptor 2
PRRT	Peptide receptor radionuclide therapy
PFS	Progression-free survival
OS	Overall survival
DMEM	Dulbecco’s modified eagle medium
FBS	Fetal bovine serum
RCP	Radiochemical purity
Radio-HPLC	Radio high-performance liquid chromatography
TFA	Trifluoroacetic acid
PBS	Phosphate-buffered saline
RT	Room temperature
7-AAD	7-amino-actinomycin D
MFI	Mean fluorescence intensity
CFDA SE	Carboxyfluorescein diacetate, succinimidyl ester
FITC	Fluorescein isothiocyanate
PI	Propidium iodide
DSB	Double-strand break
ANOVA	Analysis of variance
NHEJ	Non-homologous end-joining
